# Obesity can influence children’s and adolescents’ airway hyperresponsiveness differently

**DOI:** 10.1186/2049-6958-8-60

**Published:** 2013-09-12

**Authors:** Bruno Sposato, Marco Scalese, Maria Giovanna Migliorini, Maria Piera Riccardi, Massimo Tosti Balducci, Luigi Petruzzelli, Raffaele Scala

**Affiliations:** 1 Unit of Pneumology, Azienda Ospedaliera, “Misericordia” Hospital, Via Senese 161, 58100, Grosseto, Italy; 2 Institute of Clinical Physiology, National Research Council (CNR), Pisa, Italy; 3 Unit of Infectivology “Misericordia” Hospital, Grosseto, Italy; 4 Unit of Nuclear Medicine “Misericordia” Hospital, Grosseto, Italy; 5 Unit of Pneumology and UTIP, “S.Donato” Hospital, Arezzo, Italy

**Keywords:** Airway hyperresponsiveness, Asthma, Body mass index, Children and adolescents, Methacholine test, Obesity

## Abstract

**Background:**

Literature is still arguing about a possible relationship between airway hyperresponsiveness (AHR) and body mass index (BMI). This study aimed at evaluating the influence of BMI on AHR and pulmonary function in children and adolescents that performed a methacholine test for suggestive asthma symptoms.

**Methods:**

799 consecutive children/adolescents (535 M; mean age: 15 ± 3 yrs; median FEV_1_% predicted: 101.94% [93.46-111.95] and FEV_1_/FVC predicted: 91.07 [86.17-95.38]), were considered and divided into underweight, normal, overweight and obese. Different AHR levels were considered as moderate/severe (PD_20_ ≤ 400 μg) and borderline (PD_20_ > 400 μg).

**Results:**

536 children/adolescents resulted hyperreactive with a median PD_20_ of 366 μg [IQR:168–1010.5]; 317 patients were affected by moderate/severe AHR, whereas 219 showed borderline hyperresponsiveness. Obese subjects aged > 13 years showed a lower (p = 0.026) median PD_20_ (187μg [IQR:110–519]) compared to overweight (377 μg [IQR:204–774]) and normal-weight individuals’ values (370.5 μg [IQR:189–877]). On the contrary, median PD_20_ observed in obese children aged ≤ 13 years (761 μg [IQR:731–1212]) was higher (p = 0.052) compared to normal-weight children’s PD20 (193 μg [IQR:81–542]) and to obese adolescents’ values (aged > 13 years) (p = 0.019). Obesity was a significant AHR risk factor (OR:2.853[1.037-7.855]; p = 0.042) in moderate/severe AHR adolescents. Females showed a higher AHR risk (OR:1.696[1.046-2.751] p = 0.032) compared to males. A significant relationship was found between BMI and functional parameters (FEV_1_, FVC, FEV_1_/FVC) only in hyperreactive females.

**Conclusions:**

Obesity seems to influence AHR negatively in female but not in male adolescents and children. In fact, AHR is higher in obese teenagers, in particular in those with moderate/severe hyperresponsiveness, and may be mediated by obesity-associated changes in baseline lung function.

## Background

Many researches have shown that asthma and obesity are associated. In particular, the increase in body mass index (BMI) has been associated with an increased incidence and prevalence of asthma in pediatric population [1-4]. However, available clinical data on obesity and airway hyperresponsiveness (AHR), which is a feature of asthma, are conflicting. In fact, many studies, concerning this topic and conducted on children, have not demonstrated any relationships between BMI and airway hyperresponsiveness [5-9]. Other studies, on the contrary, have shown that a higher BMI was associated with a higher AHR [10-12]. These conflicting results may be due to the fact that, in a few studies, a diagnosis of asthma is sometimes established on symptoms reported by children’s parents. Furthermore, this may also be due to over-diagnosis of asthma because obesity-related chest symptoms may in fact mimic asthma. In addition, when a higher cut-off value is used to define a positive bronchial provocation test, asthma diagnosis may be overestimated [13] and this can obviously influence the outcome of the BMI relationship. Also, a regular use of inhaled corticosteroids, bronchodilators and anti-leukotriens may change the results in bronchial provocation tests [14,15].

Another controversial issue is the different role of BMI with regard to gender. In fact, some authors found that a higher BMI was related to a higher PC_20_ (a lower airway reactivity) in female children but not in males [6]. On the contrary, Huang et al. noted a decreased prevalence of AHR in the lowest quintile of BMI in teenage girls [10]. Another study observed that BMI was associated with AHR in school-age boys [11].

There are also controversial data on a possible influence of BMI on pulmonary function; some authors found a reduction in FEV_1_, FVC and FEV_1_/FVC with a weight increase [6,16,17], whereas the opposite was observed by other authors [18]. Besides, studies often associate children with adolescents, without taking into account that puberty can influence asthma differently [19]. Also smoking can have a negative impact on bronchial hyperreactivity [20].

Therefore, taking all this into account, the aim of our retrospective study was to assess whether there is a relationship between BMI and AHR, especially in different levels of AHR, in children and adolescents that had undergone a methacholine challenge test for suspected asthma. We also tried to assess whether smoking could interfere with the correlation between BMI and AHR. Another aim was to evaluate whether there is an association between BMI and baseline lung function. In order to limit the influence of treatment, only subjects who were not treated regularly with asthma medications were included in the study.

## Methods

### Subjects

In our retrospective study, we analyzed the results of 821 consecutive methacholine challenge tests on subjects aged < 18 years performed between 2000 and 2008 in the Pneumology Unit, “Misericordia” Hospital, Grosseto and in the Pneumology and UTIP Unit, “S. Donato” Hospital, Arezzo, Tuscany, Italy.

All subjects reported symptoms suggestive of asthma (unexplained episodes of cough and/or wheezing and/or dyspnea on exertion) associated with a normal baseline lung function and they all had performed the methacholine test in order to confirm the diagnosis of asthma. Only 799 consecutive subjects (535 M; mean age 15 ± 3yrs; median FEV_1_% 101.94 [93.46-111.95] and FEV_1_/FVC 91.07 [86.17-95.38]), were suitable for the study. Twenty-two subjects were excluded because they had not completed the test as showing a fall in FEV_1_> 10% with buffer solution, thus there were no PD_20_ values in these subjects. FEV_1_, FEV_1_/FVC, FVC, FEF_25-75,_ measured at baseline (pre-Mch test) and PD_20_FEV_1_ recorded in each bronchoprovocation test were considered for the study. Smoking habits, age, sex and BMI were also taken into account.

None of the subjects was under regular asthma treatment when the test was performed. Subjects who had taken drugs when required were asked to avoid taking any medications prior to the test: β_2_-agonist bronchodilators and inhaled or systemic corticosteroids were suspended 24 hours and 3 weeks before the test respectively, while antihistamines were suspended at least 10 days before the challenge. No subjects had suffered from airway infections or asthma exacerbations in the four weeks prior to the test. International age and sex specific cut off points for BMI were used to subdivide subjects with age < 18 years into underweight, normal or overweight-obese [21,22]. BMI was calculated by dividing the weight in kilograms by the square of height in metres (kg/m^2^). Cut-offs used to divide the cases in the various weight classes, were equivalent to adult BMI values. Children/adolescents that showed an adult corresponding value less than 18.5 or between 18.5 and 25 or between 25 and 30 or > 30 were considered as underweight, normal weight, overweight and obese, respectively.

The use of data for the purpose of the study was approved by the local Ethic Committees which did not retain necessary to obtain any informed consent from each patient because data were retrospectively and anonymously analyzed.

### Mch bronchoprovocation test

The Mch bronchoprovocation test was performed by using a dosimeter method [13]. The same instrument and method were used both in Grosseto and Arezzo. Mch sulphate was supplied by Lofarma (Milan, Italy) and administered in aerosol form using a MEFAR MB3 dosimeter (output: 9 μL/puff; MEFAR Elettromedicali Brescia, Italy) with MB2 ampoule model. The buffer solution was the first to be administered, followed by 40 μg of methacholine, increasing the doses until PD_20_FEV_1_ was obtained or until the maximum dose of Mch was reached. FEV_1_ was assessed after inhaling 40, 80, 120, 240, 400, 800, 1,600, and 2,400 μg of cumulative Mch doses, respectively. At the end of exhalation, during tidal breathing, patients inhaled Mch slowly and deeply for 5 seconds and then held their breath for 5 seconds more. The test was interrupted if a drop in FEV_1_> 10% took place with the buffer solution. The interval between two consecutive steps was 2 minutes. FEV_1_ was measured at 30 and 90 seconds after nebulization. A suitable quality of FEV_1_ was obtained at each step. No more than two maneuvers after each dose were allowed, and the highest FEV_1_ value was considered. AHR was defined by a 20% fall of FEV_1_ value from the reference one (see below) obtained with a cumulative Mch dose < 2,400 μg. Subjects who did not achieve a 20% fall in FEV_1_ with a Mch dose of 2,400 μg were regarded as normoreactive.

Subjects with PD_20_ ≤ 400 and PD_20_ > 400 μg were considered as affected by moderate to severe and borderline AHR respectively, with the aim of evaluating the effects of BMI on the different levels of AHR.

The lung function during the test was measured with a HP 47120E Pulmonary System Desk spirometer (Hewlett Packard, Waltham, Massachusetts - USA). FEV_1_ and FVC were expressed as percentages of the predicted values at baseline, whereas FEV_1_/FVC was reported only as a ratio (reference equation: CECA, 1971). PD_20_ FEV_1_ was assessed by linear interpolation of the dose–response curves. The FEV_1_ measured before administering the buffer solution was taken as baseline value, while the FEV_1_ measured after the buffer solution was used as reference value to calculate FEV_1_ fall and thus PD_20_.

### Statistical analysis

Categorical variables are expressed as number of cases and percentages. Continuous variables are expressed as mean values and standard deviations or median values and interquartile range (IQR – 25° and 75° quartiles) according to whether they are normally distributed. Nonparametric or parametric tests were performed accordingly. Comparisons of qualitative data were performed using the chi-square test, whereas comparisons of quantitative variables among underweight, overweight, normal weight and obese subjects were conducted by either the ANOVA one-way test or Kruskall-Wallis test. Moreover, the Bonferroni test was used for multiple comparisons. Assessments of any possible differences between the different categories considered (males vs females, smoking vs non-smoking and children vs adolescents) were assessed with the Kruskal Wallis and Mann Whitney tests. Associations between BMI and PD_20,_ in different categories and classes of subjects considered, were analyzed using Spearman correlation test.

Five logistic binary regression models, corrected for sex, age, smoking, FEV_1_, FVC and seasons, were applied separately in overall subjects and then in males, females, children and adolescents to evaluate if BMI was an independent AHR risk factor. In order to assess a potential different risk of BMI on AHR in the various levels of hyperresponsiveness (moderate to severe and borderline AHR), comparing it to subjects with normal reactivity, two additional logistic regression models were performed for each group considered. In these models, BMI was considered as qualitative variables (underweight, normal weight, overweight and obese).

Five linear regression models (corrected for age, sex, smoking habits and seasons) were also performed separately in overall subjects and then in males, females, children and adolescents, with the purpose of assessing a possible relationship between BMI and pulmonary function (FEV_1_, FVC, FEV_1_/FVC). β-coefficients were also calculated separately for each group in subjects with moderate/severe and borderline AHR. P < 0.05 was considered statistically significant. The statistical package SPSS (16.0) was used for analysis.

## Results

A different distribution of patients with dissimilar weight was found both in males and in females. The age was lower in underweight subjects. Also, FEV_1_, FVC and FEV_1_/FVC were lower in these subjects compared to normal-weight, overweight and obese patients (Table 1).

**Table 1:** Baseline characteristics of 799 patients

	***Underweight***	***Normal weight***	***Overweight***	***Obese***	***All***	***p***
**All subjects n. (%)**	144 (18.02)	526 (65.83)	90 (12.39)	40 (5)	799 (100)	**< 0**.**001**
**Males n. (%)**	78 (14.57)	360 (67.29)	70 (13.08)	26 (4.85)	535 (100)	**0**.**001**
**Females n. (%)**	66 (25.98)	165 (64.96)	20 (7.87)	13 (5.11)	264 (100)	
**Age (mean ± SD)**	12 ± 4*° ^#^	15 ± 3*	16 ± 2°	16 ± 2^#^	15 ± 3	**< 0**.**001**
**Children, age (mean ± SD)**	9.9 ± 2.3	11.1 ± 2*	11.8 ± 1.4*	10.7 ± 1.5	10.8 ± 1.9	**0**.**0001**
**Adolescents, age (mean ± SD)**	16.1 ± 1.3	16.5 ± 1.4	16.7 ± 1.4	16.6 ± 1.3	16.4 ± 1.4	**0**.**126**
**Smokers n. (%)**	7 (7.7%)	68 (16.2%)	17 (22.1%)	4 (12.9%)	96 (15.5%)	**0**.**069**
**Subjects with normal reactivity n. (%)**	47 (32.6%)	179 (33.7%)	31 (34.4%)	6 (17.6%)	263 (32.9%)	**0**.**279**
**Hyperrea**c**tive subjects (PD**_**20 **_**< 2400 μg) n. (%)**	97 (67.4%)	352 (66.3%)	59 (65.6%)	28 (82.4%)	536 (67.1%)	
**FEV**_**1**_**%**	99.31 [88.58-108.15]*° ^#^	102.53 [94.28-111.87]*	104.47 [94.48-114.61]°	105.41 [95.48-113.14]^#^	101.94 [93.46-111.95]	**0**.**005**
**FVC %**	90.1 [82.64-98.29]*° ^#^	96.67 [88.22-105.12]*	99.24 [90.97-109.74]°	99.71 [90.21-109.50]^#^	95.89 [87.46-104.28]	**< 0**.**001**
**FEV**_**1**_**/FVC**	93.90 [8.51-97.56]*° ^#^	90.88 [86.47-95.04]*	89.15 [84.16-93.96]°	89.23 [85.73-93.44]^#^	91.07 [86.17-95.38]	**< 0**.**001**
**BMI**	17.15 [16.28-17.91]	21.36 [19.97-22.83]	26.51 [25.65-27.76]	32.45 [31.25-35.57]	21.23 [19.15-23.61]	**< 0**.**001**

FEV_1_, forced expiratory volume in one second; FVC, forced vital capacity; BMI, body mass index. The continuous variables are median (interquartile range [IQR]) or mean ± standard deviation and categorical values are expressed as number of cases (percentage). Mean comparisons were made with the ANOVA test; median comparisons were made with the Kruskal-Wallis test; proportion comparisons were made with the χ^2^ test; *post hoc* analysis was made by the Bonferroni correction.*° ^#^ statistically significant differences between groups when they were compared.

No meaningful relationships (Spearman’s correlation) between PD_20_ and BMI were found when the subjects were considered as a whole and also when subdivided into subjects with moderate/severe and borderline AHR. No relationships were found either in males, females, smokers, non-smokers, normal weight/underweight, obese subjects, children (aged< 13) or adolescents (aged> 13) even when they were divided into moderate/severe and borderline AHR individuals. Only in overweight subjects, when considered as a whole, a significant relationship was found between PD_20_ and BMI (r= −0.267; p < 0.039) (data not shown).

**Figure 1:**
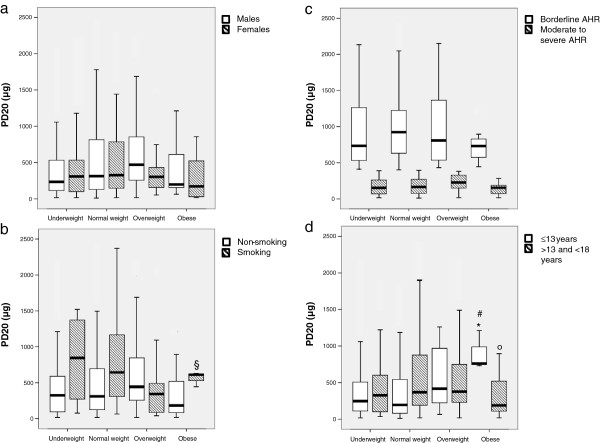
**PD**_**20**_**values measured in underweight, normal weight, overweight and obese males, females (a), smokers, non-smokers (b), moderate to severe and borderline AHR (c), and in those aged≤13 and>13 years(d).** Comparisons were made with Kruskal Wallis and Mann Whitney tests. ^§^p = 0.024: obese non-smokers in comparison with overweight non-smokers; °p = 0.026: obese subjects aged > 13 years compared to overweight and normal weight adolescents (aged > 13 years); *p = 0.052: obese children (aged ≤ 13 years) compared to children aged ≤ 13 years with normal weight; ^#^p = 0.019: obese children (aged ≤ 13 years) compared to obese adolescents (aged > 13 years). §p = 0.01.

No differences were found in PD_20_ values in underweight, overweight, normal weight and obese males and females, even when compared each other (Figure 1a). Also, when considered separately, in subjects with moderate/severe and borderline AHR (Figure 1c), median PD_20_ values were the same in each weight group. On the contrary, non-smoking obese subjects showed a lower (p = 0.024) median PD_20_ value (183.5 μg [IQR:84–519]) in comparison with non-smokers and overweight children/adolescents (443 μg [IQR:256–846]) (Figure 1b). When we considered the different age classes (Figure 1d), a lower (p = 0.026) median PD_20_ value was measured (187 μg [IQR:110–519]) in obese subjects aged > 13 years compared to overweight (377 μg [IQR:204–774]) and normal-weight (370.5 μg [IQR:189–877]) individuals. On the contrary, the median PD_20_ value measured in obese children aged ≤ 13 years (761 μg [IQR:731–1212]) was higher (p=0.052) than that observed in normal-weight children (193 μg [IQR:81–542]). Furthermore, this latter PD_20_ value, measured in obese children (aged ≤ 13years), was different even from that observed in obese adolescents (aged > 13 years) (p = 0.019) (Figure 1d).

**Figure 2:**
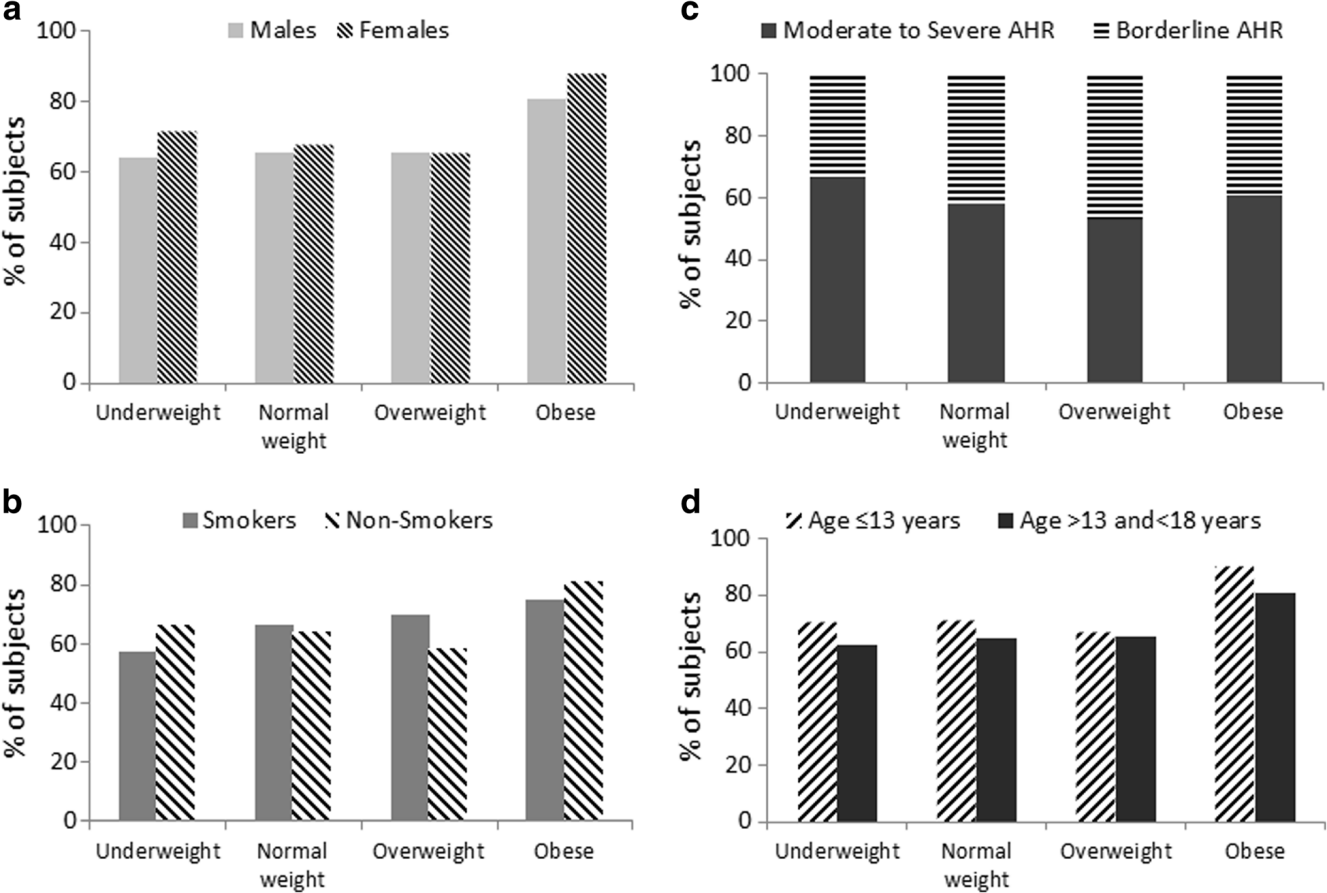
**Prevalence of subjects with airway hyperresponsiveness (PD**_**20 **_**< 2,400 μg) obtained in underweight, normal weight, overweight and obese males, females (a), smokers, non-smokers (b), moderate/severe and borderline AHR (c), and in those aged ≤ 13 and>13 years(d).** No differences among different groups and sub-groups (χ^2^ tests).

No differences were observed in AHR prevalence either in the different groups of subjects (underweight, overweight, normal-weight and obese) or in the various sub-groups (males, females, different-age groups, smokers and non-smokers) (Figure 2).

The logistic regression model (Table 2)showed that in all hyperreactive subjects the obese condition (in comparison to subjects with normal weight) resulted to be a risk factor (corrected for age, sex, smoking habits, FEV_1_, FVC and seasons) for AHR (OR: 2.653 [1.055-6.673], p = 0.038; Table 2). When subjects were analyzed for different AHR degrees, the obesity condition was a risk factor for AHR only in subjects that showed a moderate/severe AHR (OR: 2.874 [1.059-7.800], p=0.038) but not in borderline AHR individuals (2.299 [0.809-6.528], p=0.118). FVC and FEV_1_ were risk and protective factors for AHR in all AHR levels (data not reported). At this stage, we applied separate logistic regression models considering only males, females, children (aged ≤ 13 years) and adolescents (aged > 13 years) (Table 2). In females, even if not statistically significant, a higher odd ratio value in obese women was noted, especially in those with moderate/severe AHR (OR:4.380 [0.417-46.009]) showing that the risk of AHR might be higher in obese females. On the contrary, the obese condition resulted a significant risk factor for AHR (OR:2.853 [1.037-7.855]; p = 0.042) in adolescents (aged > 13 years) with moderate/severe AHR. When this logistic regression model was applied, we also noted that females’ conditions were a significant risk factor for AHR (OR:1.696 [1.046-2.751]; p = 0.032) in adolescents with moderate/severe hyperresponsiveness. This was not observed either in all subjects, when considered as a whole, or in those aged ≤ 13 years (data not shown). Furthermore, FEV_1_ resulted to be a significant protective factor from AHR whereas FVC was a risk factor in all regression logistic models applied (data not shown).

**Table 2:** **OR obtained in all subjects with AHR** (**PD**_**20**_ <**2400 μg**) **and in those with only moderate**/**severe** (**PD**_**20**_ ≤ **400 μg**) **or borderline AHR** (**PD**_**20**_ >**400 μg**) **in comparison to subjects with normal reactivity**

	**All AHR subjects (PD**_**20 **_**< 2400 μg)**			**Subjects with moderate/severe AHR (PD**_**20 **_**< 400 μg)**			**Subjects with borderline AHR (PD**_**20**_**>400 μg)**		
	OR	95% CI	p	OR	95% CI	p	OR	95% CI	p
**All underweight subjects**	0.814	0.522-1.268	0.362	0.800	0.478-1.338	0.396	0.815	0.463-1.434	0.478
**All overweight subjects**	0.976	0.598-1.594	0.955	0.876	0.490-1.566	0.654	0.958	0.534-1.721	0.887
**All obese subjects**	2.653	1.055-6.673	**0**.**038**	2.874	1.059-7.800	**0**.**038**	2.299	0.809-6.528	0.118
**Underweight males**	0.609	0.336-1.104	0.102	0.525	0.265-1.041	0.065	0.559	0.261-1.199	0.135
**Overweight males**	1.011	0.577-1.772	0.969	0.809	0.413-1.586	0.537	0.992	0.515-1.911	0.980
**Obese males**	2.404	0.864-6.691	0.093	2.480	0.804-7.649	0.114	2.208	0.697-6.995	0.178
**Underweight females**	1.221	0.607-2.456	0.576	1.487	0.645-3.429	0.352	1.399	0.573-3.417	0.461
**Overweight females**	0.766	0.272-2.159	0.614	1.250	0.383-4.079	0.711	0.572	0.138-2.372	0.441
**Obese females**	4.058	0.439-37.557	0.217	4.380	0.417-46.009	0.118	2.863	0.210-39.048	0.430
**Underweight subjects (age≤13yrs)**	0.788	0.396-1.566	0.496	0.616	0.276-1.374	0.236	1.078	0.442-2.629	0.869
**Overweight subjects(age≤13yrs)**	1.244	0.326-4.749	0.749	1.250	0.219-7.142	0.802	3.066	0.568-16.551	0.193
**Obese subjects (age≤13yrs)**	1.952	0.177-21.516	0.585	1.565	0.069-35.417	0.778	3.934	0.313-49.423	0.289
**Underweight subjects (age>13)**	0.828	0.447-1.537	0.550	0.986	0.474-2.052	0.970	0.744	0.335-1.652	0.468
**Overweight subjects(age>13)**	0.935	0.547-1.597	0.805	0.851	0.455-1.593	0.615	0.802	0.423-1.522	0.500
**Obese subjects (age>13)**	2.390	0.933-6.125	0.070	2.853	1.037-7.855	**0**.**042**	1.580	0.510-4.891	0.428

Five different logistic regression models (respectively for overall, males, females, children and adolescents) were applied and corrected for sex (where appropriate), age, smoking habits, FEV_1_, FVC and seasons. Odd ratios were also calculated in subjects with moderate/severe and borderline AHR. Underweight, overweight and obese subjects were compared to those with normal weight. Statistically significant values are in bold.

In the attempt to find possible explanations for the above-described associations between BMI and AHR, we applied the regression linear models in order to connect lung function and BMI, adjusted for age, season, smoking habits and sex, when possible (Table 3). No relationships between BMI and pulmonary function were observed either in males or in the different age classes. We only noted a significant variation of FEV_1_ and FVC for a BMI unit increase in females with AHR but not in those with normal bronchial reactivity (see Table 3 for values in bold).

**Table 3:** **Relationship between lung function** (**measured at baseline**, **before the Mch test**) **and BMI** (**linear regression models**) **corrected for age**, **seasons**, **smoking habits and sex when appropriate**

	**All AHR subjects (PD**_**20**_**<2400 μg)**		**Subjects with moderate**/**severe AHR(PD**_**20**_**<400 μg)**		**Subjects with borderline AHR (PD**_**20**_**>400 μg)**		**Subjects with normal reactivity**	
	β	p	β	p	β	p	β	p
FVC% (**All**)	0,161	0,123	0,167	0,293	0,239	0,217	0,227	0,353
FEV_1_% (**All**)	−0,077	0,410	−0,105	0,473	−0,168	0,326	−0,087	0,690
FEV_1_/FVC (**All**)	0,099	0,383	0,131	0,463	0,132	0,523	0,187	0,469
FVC% (**Males**)	0.053	0.730	0.086	0.611	0.107	0.748	0.158	0.615
FEV_1_% (**Males**)	0.010	0.942	−0.007	0.962	−0.056	0.851	−0.033	0.905
FEV_1_/FVC (**Males**)	0.001	0.994	0.035	0.855	0.046	0.895	0.126	0.707
FVC% (**Females**)	0.411	**0**.**027**	0.934	**0**.**035**	0.390	**0**.**015**	0.240	0.471
FEV_1_% (**Females**)	−0.348	**0**.**036**	−0.849	**0**.**036**	−0.315	**0**.**024**	−0.095	0.744
FEV_1_/FVC (**Females**)	0.290	0.147	0.882	0.059	0.075	0.665	0.195	0.575
FVC% (**Age** ≤**13**)	−0,060	0,725	−0,038	0,803	0,094	0,927	−0,177	0,713
FEV_1_% (**Age** ≤**13**)	0,112	0,469	0,099	0,485	−0,054	0,953	0,225	0,611
FEV_1_/FVC (**Age** ≤**13**)	−0,189	0,314	−0,127	0,470	−0,223	0,825	−0,173	0,720
FVC% (**Age**>**13**)	0,174	0,261	0,260	0,327	0,124	0,525	0,144	0,561
FEV_1_% (**Age**>**13**)	−0,119	0,399	−0,193	0,438	−0,084	0,633	−0,023	0,916
FEV_1_/FVC (**Age**>**13**)	0,108	0,523	0,228	0,444	0,015	0,944	0,070	0,790

## Discussion

This research highlights that obesity can influence airway hyperresponsiveness especially in asthmatic adolescents that show a moderate/severe AHR to the methacholine challenge test but not in children whose weight increase, on the contrary, might have a positive effect on bronchial hyperresponsiveness. Furthermore, obesity is a significant risk factor for AHR in females rather than in male adolescents and children. Our data show that this effect may be mediated in females by an influence of BMI on pulmonary function.

As already said, an important and original finding of this study is the different influence of BMI in different age classes, especially in children and adolescents. In the first class, no significant negative effects of BMI were observed. On the contrary, weight increase seems to be related to a higher PD_20_ value. In fact, a higher PD_20_ was observed in obese children, in comparison to those with normal weight and even with obese adolescents. Some authors observed that a higher BMI was not a risk factor for airway hyperresponsiveness in either boys or girls aged ≤ 12 years [7,8], whereas others, like us, found that BMI was even positively associated with PC_20_ in children aged 7–12 years and with increasing spirometric values [6]. This suggested a possible protective effect of weight in children. The discovery of a positive association between PC_20_ and BMI was still somewhat puzzling. No explanation has been found in literature. This positive relationship between BMI and AHR may be simply due to the fact that several obese children, rather than those with normal weight, may be not asthmatics because in reality obesity-related chest symptoms may be mistaken for asthma [5]. On the contrary, a lower PD_20_ value was measured in obese adolescents aged > 13 years when compared to overweight and normal weight individuals. Also other studies showed that BMI was a significant AHR predictor in adolescents [10,16]. These results may be justified by the fact that the effects of obesity on AHR may occur only after a certain period of time from its onset. In fact, children who had a persistent high BMI during childhood, had a significantly increased risk of having AHR when they were 8 years old, whereas those that normalized their BMI from 6 to 7 years, did not have an increased AHR risk at the age of 8 [12]. Therefore, the possible negative effects induced by proinflammatory molecules (leptin, tumor necrosis factor α (TNF-α), interleukin 6 (IL-6), transforming growth factor β1 (TGF-β1), C-reactive protein, adiponectin and resistin) on AHR and in general on asthma [23,24], may be delayed in time, as they need a latency period before showing their effects.

Another reason for this difference between children and adolescents may be due to pubertal sex hormone differences. In particular, this difference should regard females because they have a precocious puberty in comparison with males. In fact, as already said, we found a lower PD_20_ in obese adolescents if compared to obese children. Furthermore, an enhanced AHR risk was found in adolescents with moderate/severe AHR. The risk evaluation in the two sexes showed that it was 38% greater in females when compared to males. This means that obesity influences only female adolescents and not male adolescents or children, which is perfectly in line with what was assessed in another study of ours [20] and in previous researches [23,25,26] where a higher AHR risk was observed only in adult females. Female adolescents become adults more precociously than males. Therefore, BMI influences AHR only when it interacts with some features that are acquired during adulthood. Sex hormone differences may play an important role in this dissimilar behaviour. In particular estrogens may play a role in modulating the relationship between BMI and asthma. In fact, the estrogens level increases with obesity favouring an early menarche in women and a delay in the onset of puberty in men [23]. It was found that the prevalence of asthma and the association between BMI with the severity of the disease were greater in women with an early menarche [27,28]. Furthermore, it seems that estrogens and progesterone may modify the inflammatory response to favour a Th2 response [23]. β-estradiol enhances eosinophil adhesion to human mucosal micro-vascular endothelial cells and may induce degranulation (unlike the testosterone effect), whereas progesterone increases bronchial eosinophilia and enhances bronchial responsiveness [29,30]. In addition, this different hormonal pattern determines a greater subcutaneous fat distribution, while in males it causes a higher visceral adipose tissue localization. Subcutaneous abdominal fat appears to increase the risk of airway hyperreactivity, while visceral abdominal fat seems not to be associated with AHR [31]. In particular, a higher level of bronchial hyperresponsiveness (after a hypertonic saline challenge test) was associated with a gynoid fat mass in females [32]. Subcutaneous fat, rather than visceral fat, produces a higher amount of leptin (with greater values in females compared to males) which may be responsible for asthma worsening in obese women [23,33,34]. In fact, leptin may induce airway inflammation in asthmatics. A relationship between circulating leptin levels and risk of asthma development was observed in females [34]. Recently, an increased neutrophilic inflammation was found only in asthmatic obese females [25]. Another research observed that a gynoid fat mass is associated with a lower concentration of airway eosinophils in females [25]. Therefore, BMI may influence AHR differently in males and females with a dissimilar inflammatory pattern induced by obesity. Likely, a higher production of leptin from subcutaneous adipose tissue, which is typical of females, promotes T-helper type 1 cell differentiation and increases activation of neutrophils via tumour necrosis factor α [35].

An alternative explanation for our findings could be either the purely mechanical effect of weight on pulmonary function, and consequently on AHR, or an indirect effect of systemic inflammation (as already pointed out) on bronchial reactivity via a lung function impairment. In fact, we observed a FEV_1_ reduction and an increase in FVC for unit of increase in BMI in female adolescents who already showed an association between BMI and AHR. Furthermore, we also found that an increase in FEV_1_ and FVC resulted to be significantly protective and risk factors for AHR respectively in all classes of subjects considered. This means that a BMI increase determines functional alterations consistent with an obstructive pattern in female adolescents. This is in accordance with a previous study of ours, carried out on adults, where we found a significant reduction of FEV_1_/FVC ratio only in females with moderate AHR [20]. Also other researchers found a FEV_1_/FVC reduction both in obese children [6] and adults [36], though this was not confirmed by other authors [32,37]. This more significant BMI-induced obstruction could consequently influence AHR. In our study, FEV_1_ increase was an AHR protective factor, thus suggesting that a reduction of lung function may lead to AHR. An excess soft tissue weight compressing the thoracic cage, a fatty infiltration of chest wall and an increase in pulmonary blood volume, could contribute to determine a reduction in lung volumes for a mechanical effect especially in females [26,36,38]. This is associated with an impairment in the lung inflation-induced airway distensibility and a reduction in peripheral airway diameter, which, over time, may perturb smooth muscle function thus increasing both airway obstruction and AHR [23]. BMI seems not to influence lung function in males while it appears to have a significant impact in females. It is likely that the smaller airway calibre in females may be influenced by BMI-induced obstruction in a more pronounced way than in males. This is supported by other studies which also observed a greater effect of adipose tissue on females’ lung function compared to males [25,39].

In children, as already said, no relationships between BMI and lung function was found. However, other researchers reported contradictory results. Some authors found reduced values of FEV_1_, FVC and FEV_1_/FVC in healthy [17] and asthmatics overweight and obese children [6,16] while others reported spirometric measurement increases in parallel with body weight and BMI [6,18]. These conflicting results may be due to an influence of growth variability in children. A possible negative effect of obesity on lung function may be hidden by a positive effect due to growth.

Another interesting result was the dissimilar influence of BMI on the two AHR different levels considered. The logistic regression models showed a diverse relationship between BMI and the different levels of AHR (moderate/severe and borderline). A significant risk for AHR was only found in subjects with moderate/severe hyperresponsiveness and not in those with borderline reactivity. The absence of any association between BMI and AHR in borderline hyperresponsiveness is certainly due to the fact that a great proportion of subjects belonging to this group ended up by not being asthmatics. In fact, high values of PD_20_ or PC_20_, in case of suspected asthma (like in our patients), make an asthma diagnosis less probable [13]. Bronchial hyperresponsiveness is a characteristic of asthma and inflammation is related to AHR. In fact, some researchers found that nitric oxide levels significantly increase with the increasing ofbronchial hyperresponsiveness in asthmatics [40,41]. Therefore, it seems that obesity interacts only with subjects affected by asthma and especially with those suffering from a more severe form. Obesity is considered as an inflammatory status, because proinflammatory molecules, expressed by adipose tissue such as leptin, TNF-α, IL-6, TGF- β1, adiponectin and C-reactive protein, are increased in obese subjects [23,25], especially in females (mainly C-reactive protein and leptin) [20]. Thus, obesity, with additional inflammation, may have a variable role, like several other environmental events (*i.e.* allergens, respiratory infections, and treatment), in increasing the pre-existing bronchial hyperreactivity [13]. In confirmation of this, weight loss, through bariatric surgery, produces significant improvements in exhaled nitric oxide in obese asthmatic patients and in AHR, especially in those with normal serum IgE levels [42,43].

## Conclusions

Obesity seems to be a risk factor for a greater airway hyperresponsiveness in female adolescents rather than in males and above all in children. Furthermore, this risk appears to involve only subjects with moderate/severe hyperresponsiveness and not those with borderline AHR. The relationship between BMI and AHR may be mediated by obesity-associated changes in baseline lung function.

## Competing interests

The authors declare that they have no competing interests.
